# Systemic administration of induced pluripotent stem cell-derived mesenchymal stem cells improves cardiac function through extracellular vesicle-mediated tissue repair in a rat model of ischemic cardiomyopathy

**DOI:** 10.1016/j.reth.2024.12.008

**Published:** 2024-12-31

**Authors:** Ryo Kawasumi, Takuji Kawamura, Kizuku Yamashita, Yuji Tominaga, Akima Harada, Emiko Ito, Maki Takeda, Shunbun Kita, Iichiro Shimomura, Shigeru Miyagawa

**Affiliations:** a Department of Cardiovascular Surgery, Osaka University Graduate School of Medicine, Suita, Osaka, Japan; b Department of Metabolic Medicine, Graduate School of Medicine, Osaka University, Osaka, Japan; c Department of Adipose Management, Graduate School of Medicine, Osaka University, Osaka, Japan

**Keywords:** Ischemic cardiomyopathy, Pluripotent stem cell, Extracellular vesicles, microRNA, Left ventricular ejection fraction

## Abstract

**Introduction:**

Systemic administration of induced pluripotent stem cell-derived mesenchymal stem cells (iPS-MSCs) has a therapeutic effect on myocardial ischemia. However, the therapeutic mechanism underlying systemic iPS-MSC-based therapy for ischemic cardiomyopathy (ICM) remains unclear. We investigated the therapeutic effects of iPS-MSCs through extracellular vesicle (EV)-mediated tissue repair in a rat model of ICM.

**Methods:**

A rat ICM model was created by left anterior descending coronary artery ligation. iPS-MSCs were administered intravenously every week for four weeks in the iPS-MSC group, whereas saline was administered to the control group. Alix, a protein involved in the biogenesis of EVs, was knocked down, and Alix-knockdown iPS-MSCs were administered to the siAlix group. We analyzed sequential cardiac function using echocardiography, histological analysis, cell tracking analysis with fluorescent dyes, and comprehensive RNA sequencing of the border zone of the myocardium after treatment.

**Results:**

Left ventricular ejection fraction (LVEF) was significantly improved in the iPS-MSC group compared with that in the control group. In the siAlix group, LVEF was significantly lower than that in the iPS-MSC group. Histological analysis showed a significant decrease in fibrosis area and significant increase in microvascular density in the iPS-MSC group. A cell-tracking assay revealed iPS-MSC accumulation in the border zone of the myocardium during the acute phase. Comprehensive microRNA sequencing analysis revealed that EVs from iPS-MSCs contained miRNAs associated with anti-fibrosis and angiogenesis. Gene ontology analysis of differentially expressed genes in myocardial tissue also showed upregulation of pathways related to antifibrosis and neovascularization and downregulation of pathways linked to inflammation and T-cell differentiation.

**Conclusions:**

Systemic administration of iPS-MSCs improved cardiac function through EV-mediated angiogenetic and antifibrotic effects in an ICM, suggesting the clinical possibility of treating chronic heart failure.

## Graphical abstract

**Figure:**
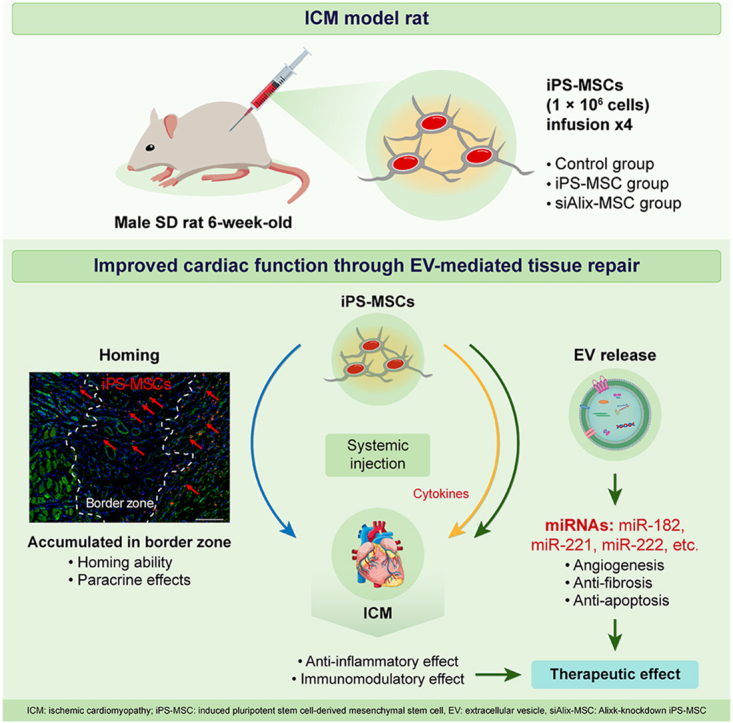

## 1 Introduction

Ischemic cardiomyopathy (ICM) is a condition caused by extended coronary artery disease that eventually leads to significant left ventricular systolic function impairment [1]. We have previously reported that cardiac regenerative therapy using autologous skeletal myoblast sheet and induced pluripotent stem cell-derived cardiomyocytes sheet has been used in a clinical setting and improved event-free survival in patients with ICM [2,3]. However, the feasibility of these therapies was limited in terms of surgical invasiveness and adverse effects of immunosuppressant therapy. Therefore, novel cell therapies that can be administered intravenously and engrafted without immunosuppressants are required.

Mesenchymal stem cells (MSC) are multipotent stem cells that exert therapeutic effects in various diseases through angiogenetic, anti-inflammatory, and immunomodulatory effects, as well as a homing ability to the damaged tissue sites. Extracellular vesicles (EVs), which are lipid bilayer vesicles secreted from cells, are known to be a key therapeutic factor in the physiologic function of MSCs [[4], [5], [6]]. Micro RNAs, the cargo of EVs, are a family of non-coding RNAs that are involved in regulating the expression of target genes and mediating intracellular communication [7]. MSCs can be derived from various tissues, including adipose tissue, bone marrow, Wharton's jelly, dental pulp, skin, and peripheral blood [8]. Induced pluripotent stem cell-derived MSCs (iPS-MSCs) are a novel type of stem cell derived from human induced pluripotent stem cells, which are considered to be promising cell sources of MSC production because of their inexhaustibility, consistent quality, and unlimited proliferation capacity [9,10]. IPS-MSCs are expected to be ideal cell sources because of their independence from the donor's tissue quality, unlike other tissue-derived MSCs [10]. A cell therapy using iPS-MSCs has also been preclinically applied to various disorders in regenerative therapy and exhibited more significant therapeutic efficacy than bone marrow-derived MSCs (BM-MSC) [11,12]. Some studies have shown that systemic iPS-MSC administration improves cardiac function in myocardial ischemia [13,14]. However, little information is available on the therapeutic mechanism of systemic iPS-MSC-based therapy in ICM. Therefore, in this study, we hypothesized that systemic administration of iPS-MSCs improves cardiac function through EV-mediated tissue repair in a rat model of ICM.

## 2 Methods

### 2.1 Cell culture and induction of iPS-MSCs

**Fig. 1:**
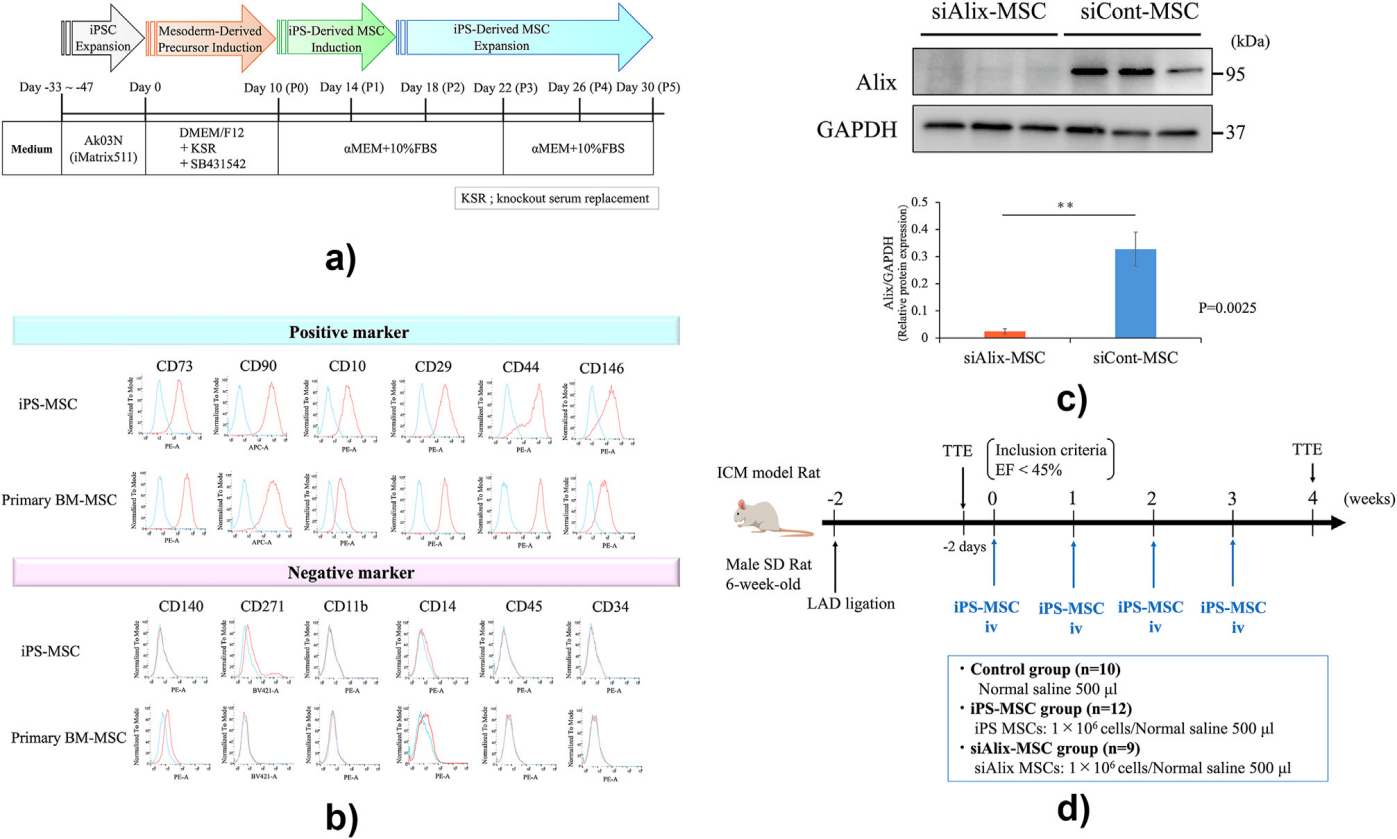
**Preparation of iPS-MSCs and study protocol.** (a) Protocol for iPS-MSC differentiation. (b) Characterization of iPS-MSCs and primary BM-MSCs using flow cytometry. (c) iPS-MSCs transfected with or without Alix siRNA were subjected to Western blot analysis. (d) Experimental design for sequential administration of iPS-MSCs to a rat ICM model. iPSC, induced pluripotent stem cell; iPS-MSC, induced pluripotent stem cell-derived mesenchymal stem cell; DMEM/F12, Dulbecco's modified Eagle's medium and Ham's F-12; KOSR, knockout serum replacement; αMEM +10 % FBS, alpha-modified Eagle minimum essential medium combined with 10 % fetal bovine serum; BM-MSC, bone marrow-derived mesenchymal stem cell; ICM, ischemic cardiomyopathy; SD rat, Sprague-Dawley rat; LAD, left ascending coronary artery; TTE, transthoracic echocardiography; EF, ejection fraction; siAlix-MSC, Alix-knockdown induced pluripotent stem cell-derived mesenchymal stem cell. siCont-MSCs and iPS-MSCs without Alix knockdown Data are presented as mean ± SD. ∗∗p < 0.01 between groups, by Student's t-test.

We used a clinical-grade human iPSC line, QHJI14s04, established from peripheral blood mononuclear cells and provided by the Center for iPS Cell Research and Application, Kyoto University (Kyoto, Japan). QHJI14s04 cells were seeded on iMatrix511 (Nippi, Tokyo, Japan)-coated dishes and cultured in stem-fit Ak03 N (Ajinomoto, Tokyo, Japan). The iPS-MSCs were differentiated according to a previously published method (Fig. 1a) [15]. Briefly, iPSCs were grown in a medium comprising Dulbecco's modified Eagle's medium and Ham's F-12 (DMEM/F-12; Nacalai Tesque, Kyoto, Japan), supplemented with 20 % knockout serum replacement (KOSR). After cell confluence, the medium was changed to KOSR medium supplemented with 10 μM SB341542, a transforming growth factor-β pathway inhibitor. After 10 d, the cells were transferred into an alpha-modified Eagle minimum essential medium combined with 10 % fetal bovine serum (⍺MEM + 10 % FBS; Nacalai Tesque), and differentiation was then completed. Cells were analyzed by flow cytometry using a fluorescence-activated cell sorting (FACS) Canto II system (BD Biosciences, NJ, USA). Primary BM-MSCs (PromoCell, Heidelberg, Germany) were used as controls. Data analysis was performed using FlowJo (Tree Star, OR, USA).

### 2.2 siRNA knockdown of Alix expression

The siRNA transfection was performed per the manufacturer's protocol. Briefly, iPS-MSCs (1.0 × 10^6^ cells) were transfected with Alix (PDCD6IP) siRNAs (Silencer. Select; Thermo Fisher Scientific, Waltham, MA, USA) using Lipofectamine RNAiMAX Reagent (Thermo Fisher Scientific) and seeded into 6-well plates. After 48 h of incubation, cells were detached and injected for animal procedures.

### 2.3 Antibodies

The following primary antibodies were used: mouse monoclonal anti-ALIX (ab117600; Abcam) and mouse monoclonal GPADH antibody (sc-47724; Santa Cruz Biotechnology). The secondary antibody used was horseradish-peroxidase-conjugated (HRP-conjugated) sheep anti-mouse IgG (NA9310-1 ML; Cytiva, Tokyo, Japan).

### 2.4 Western blotting

After siRNA transfection, whole-cell lysates (WCL) were prepared in RIPA buffer. WCL with SDS sample buffer were loaded onto Mini Protean TGX Precast Gel (Bio-Rad, Hercules, CA, USA) for SDS-PAGE and transferred onto the PVDF transfer membrane (0.2 μm pore size; Thermo Fischer Scientific, MA, USA). The membranes were blocked using the Blocking One solution (Nacalai Tesque) and incubated with primary antibodies diluted by Can Get Signal Solution 1 (TOYOBO, Osaka, Japan) overnight at 4 °C. After washing with Tris-buffered saline and Tris-buffered saline with Tween, secondary antibodies diluted in Can Obtain Signal Solution 2 were added and incubated for 1 h at room temperature. ECL Prime Western Blotting Detection Reagent (Cytiva) was added, and a chemiluminescent signal was detected using a ChemiDoc MP imaging system (Bio-Rad). Data analysis and visualization were performed using the Image Lab software (Bio-Rad).

### 2.5 Establishment of an ICM rat model

Six-week-old male Sprague–Dawley rats (Japan SLC, Inc. Shizuoka, Japan) were anesthetized using 3 % isoflurane, intubated, and mechanically ventilated. A left mini-thoracotomy was performed, and the left anterior descending (LAD) coronary arteries were ligated [16]. After ligation, the chest was closed, and the endotracheal tube was removed after restoring spontaneous respiration. Two weeks after induction of myocardial infarction, an echocardiography was performed to confirm the establishment of the model, and rats with a preserved systolic fraction (ejection fraction >45 %) were excluded.

### 2.6 iPS-MSC administration

iPS-MSCs were suspended in sterile physiological saline (0.5 mL) and administered intravenously through the internal jugular vein. Cell infusion was performed every week, four times in all. The rats were divided into three groups: iPS-MSCs, control, and siAlix-MSCs. Sterile physiological saline (0.5 mL) was administered to the control group. In the siAlix-MSC group, Alix knockdown iPS-MSCs were produced as previously described and administered in the same manner.

### 2.7 Transthoracic echocardiography

Transthoracic echocardiography was performed 2 weeks after LAD ligation and 5 weeks after the initial iPS-MSC administration to evaluate cardiac function under anesthesia using Vivid-I (GE Healthcare, Little Chalfont, UK). The left ventricular end-diastolic diameter (LVDd, mm) and the left ventricular end-systolic diameter (LVDs, mm) were measured, and the LVEF (%) was calculated using the Teichholz method.

### 2.8 Histopathological and immunohistochemical analyses

The hearts were fixed in 10 % formalin and embedded in paraffin. Picrosirius Red staining was performed to evaluate cardiac fibrosis. The fibrotic area was calculated as the ratio of the total fibrotic area to the total left ventricular area using the MetaMorph software (version 7.10.3). Angiogenesis in the border zone was evaluated using immunohistochemical staining for vWF. Sections were visualized using diaminobenzidine and counterstained with hematoxylin. Capillary densities were calculated as the average number of vWF-positive vessels per high-power field over four fields.

### 2.9 Cell tracking analysis of iPS-MSCs after systemic administration

The distribution and localization of cells in the organs were evaluated by fluorescence imaging using CellTracker probes (Thermo Fisher Scientific). Before iPS-MSC administration, cells were stained with CellTacker dye per the manufacturer's protocol. Rats were sacrificed, and organs, including the heart, lungs, liver, and spleen, were excised 24 h after cell administration. The organ samples were fixed in 4 % paraformaldehyde (Nacalai Tesque), frozen in liquid nitrogen, and cryosectioned. Immunofluorescence staining was performed using primary and secondary antibodies. Actin filaments were stained with Alexa Fluor 488 phalloidin (Thermo Fisher Scientific), and cell nuclei were counterstained with Hoechst 33342 (Dojindo, Kumamoto, Japan). The sections were visualized under a BZ-X810 microscope (Keyence, Osaka, Japan).

### 2.10 Total RNA isolation

Total RNA was extracted from the border zone of the rat heart tissue and reverse-transcribed into cDNA. Total RNA was isolated using an RNeasy kit (Qiagen, Hilden, Germany) per the manufacturer's instructions.

### 2.11 EV extraction and miRNA isolation

iPS-MSCs were cultured in a xeno-free MSC culture medium (CiMS-BM; Cell Science & Technology Institute, Inc. Miyagi, Japan). The conditioned medium was collected after a 48-h incubation and centrifuged at 2000×*g* for 10 min at 4 °C and filtered by a vacuum filter with a 0.45 μm pore size (Nalgene Rapid-Flow Sterile Single Use Vacuum Filter Units; Thermo Fisher Scientific) to remove the cell debris. The supernatant was mixed with extra polyethylene glycol and ultracentrifuged at an average of 140,000×*g* for 2 h at 4 °C [17]. Subsequently, the EV pellet was washed with Dulbecco's PBS (Nacalai Tesque) at an average of 140,000×*g* for 2 h at 4 °C (S55A2 rotor; Eppendorf Himac Technologies, Ibaraki, Japan).

### 2.12 Comprehensive RNA and miRNA sequencing

Comprehensive RNA and microRNA sequences were obtained from the NGS Core Facility of the Genome Information Research Center at the Research Institute for Microbial Disease, Osaka University (Osaka, Japan). Sequencing was performed using an Illumina NovaSeq 6000 platform. The quality of raw paired-end sequence reads was evaluated using FastQC (version 0.11.7). Low-quality (<20) bases and adapter sequences were trimmed using the Trimmomatic software (Version 0.38), and the trimmed reads were aligned to the reference genome using the RNA-seq aligners HISAT2 (Version 2.1.0) and STAR (Version 2.7.4). The.bam files were used to estimate the abundance of mapped reads using Feature Counts (version 1.6.3). Raw read counts were normalized to transcripts per million. Pearson's correlation coefficients of the normalized counts were calculated to evaluate the correlation between the samples. Histograms and pair plots of normalized counts were generated using stats (Version 3.6.1) and gplots (Version 3.0.1.1) R packages. Heatmaps were created from the Z-scores of the normalized counts using the stats (Version 3.6.1) and gplots (Version 3.0.1.1) R packages. DEGs were identified with thresholds of |log2FC (Fold Change)| > 1 and adjusted *p*-value <0.05 using the Benjamini-Hochberg method. GO enrichment analysis of DEGs was performed using GOATOOLS (version 1.1.6). The miRNA targets were predicted using miRDB (http://mirdb.org/).

### 2.13 Statistical analysis

The data are expressed as the mean ± standard deviation (SD). Significance was determined using Student's t-test (2-tailed) to compare two groups. Differences among three groups were evaluated using one-way analysis of variance with a pot-hoc comparison. All statistical analyses were performed using JMP version 16.2.0 software (SAS Institute Inc., NC. USA). Significance was set at *p* < 0.05.

## 3 Results

### 3.1 Characteristics of differentiating iPS-MSCs

Flow cytometric analysis of iPS-MSCs revealed the positive expression of typical MSC markers, including CD73, CD90, CD10, CD29, CD44, and CD146, showing the same pattern as primary BM-MSCs (Fig. 1b). Flow cytometric analysis also showed negative expression of the macrophage and monocyte markers CD11b and CD14, as well as the hematopoietic markers CD45 and CD34.

### 3.2 Preparation of Alix-knockdown iPS-MSCs

Alix is an auxiliary protein of the endosomal sorting complex required for transport (ESCRT) and is involved in the packaging of miRNAs in EVs [18,19].

Alix knockdown in iPS-MSCs was performed using Lipofectamine RNAiMAX, a previously reported method [20,21]. Western blot analysis confirmed that Alix expression was successfully decreased in the Alix-knockdown iPS-MSC group (siAlix-MSCs) compared with that in the control group (siCont-MSCs) (Fig. 1c).

### 3.3 iPS-MSCs improve cardiac function in an ICM rat model

**Fig. 2:**
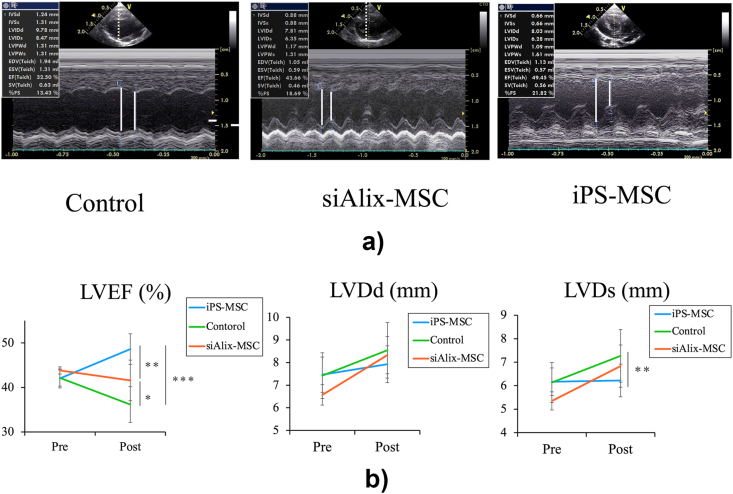
**Systemic administration of iPS-MSCs improves cardiac function in a rat ischemic cardiomyopathy model.** (a) Representative images of M-mode echocardiography in the control group, iPS-MSC group, and siAlix-MSC group. (b) Ejection fraction (EF), left ventricular end-diastolic diameter (LVDd), and left ventricular end-diastolic diameter (LVD) before and 1 week after sequential administration of cells in each group (control group, n = 10; iPS-MSC group, n = 12; siAlix-MSC group; n = 9). Data are presented as mean ± SD. ∗*p* < 0.05; ∗∗*p* < 0.01; ∗∗∗*p* < 0.001 between groups, by one-way analysis of variance with post hoc Turkey's multiple comparisons.

This protocol is illustrated in Fig. 1d. After sequential iPS-MSC administration, left ventricular ejection fraction (LVEF) was significantly improved in the iPS-MSC group compared with that in the control group (48.6 ± 3.4 % versus 36.1 ± 4.0 %, *p* < 0.0001) (Fig. 2a and b). However, the therapeutic effect of iPS-MSCs in cardiac function improvement was suppressed, and LVEF was significantly decreased in the siAlix-MSC group compared with that in the control group (41.5 ± 4.5 % versus 48.6 ± 3.4 %, *p* = 0.0019). The enlargement of left ventricular end-systolic diameter (LVDs, mm) was significantly suppressed in the iPS-MSC group compared with that in the control group (6.22 ± 0.69 versus 7.26 ± 1.19, *p* = 0.018).

### 3.4 iPS-MSCs attenuate myocardial fibrosis and promote angiogenesis in the infarct border zone

**Fig. 3:**
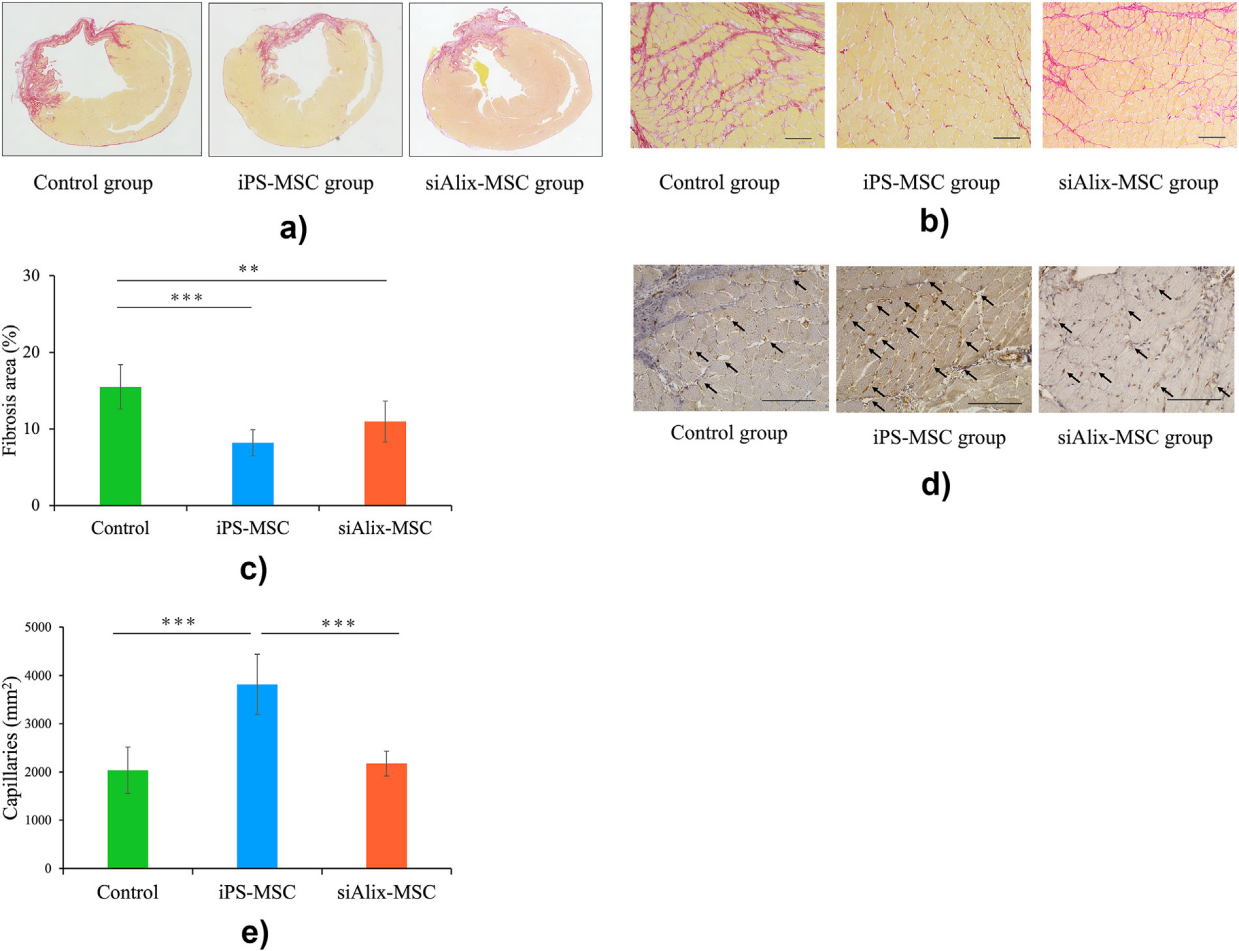
**iPS-MSCs attenuate cardiac fibrosis and promote angiogenesis in the border zone of the myocardium.** (a) Representative images of fibrosis of the whole left ventricle with picrosirius red staining in the control group, iPS-MSC group, and siAlix-MSC group. (b) Representative images of fibrosis in the border zone of the myocardium. Scale bar, 100 μm. (c) Quantification of the fibrotic area in each group (control group, n = 10; iPS-MSC group, n = 12; siAlix-MSC group; n = 9). (d) Representative images of angiogenesis in the border zone of the myocardium with immunohistochemical staining of von Willebrand factor (vWF) in each group. Arrows indicate vWF-positive capillaries. Scale bar, 100 μm. (e) Quantification of capillaries calculated by the number of vWF-positive vessels per high power field as an average over four fields. Data are presented as mean ± SD. ∗*p* < 0.05; ∗∗*p* < 0.01; ∗∗∗*p* < 0.001 between groups, by one-way analysis of variance with post hoc Turkey's multiple comparisons.

Picrosirius red staining of the heart revealed that myocardial fibrosis was significantly attenuated, especially in the infarct border zone, in the iPS-MSC group compared with that in the control group (Fig. 3a and b). The fibrotic area (%) determined from the picrosirius red staining of the whole heart was significantly decreased in the iPS-MSC group compared with that in the control group (8.18 ± 1.7 % versus 15.49 ± 2.91 %, *p* < 0.0001) (Fig. 3c). Immunohistochemical staining of the von Willebrand factor (vWF) in the infarct border zone revealed the promotion of angiogenesis in the iPS-MSC group compared with that in the control group (3813.9 ± 624.9/m^2^ versus 2037.7 ± 478.8/m^2^, *p* < 0.0001) (Fig. 3d and e). However, it was significantly decreased in the siAlix-MSC group compared with that in the iPS-MSC group (2174.1 ± 259.1/m^2^, *p* < 0.0001).

### 3.5 iPS-MSCs migrate into the infarct border zone after systemic administration

**Fig. 4:**
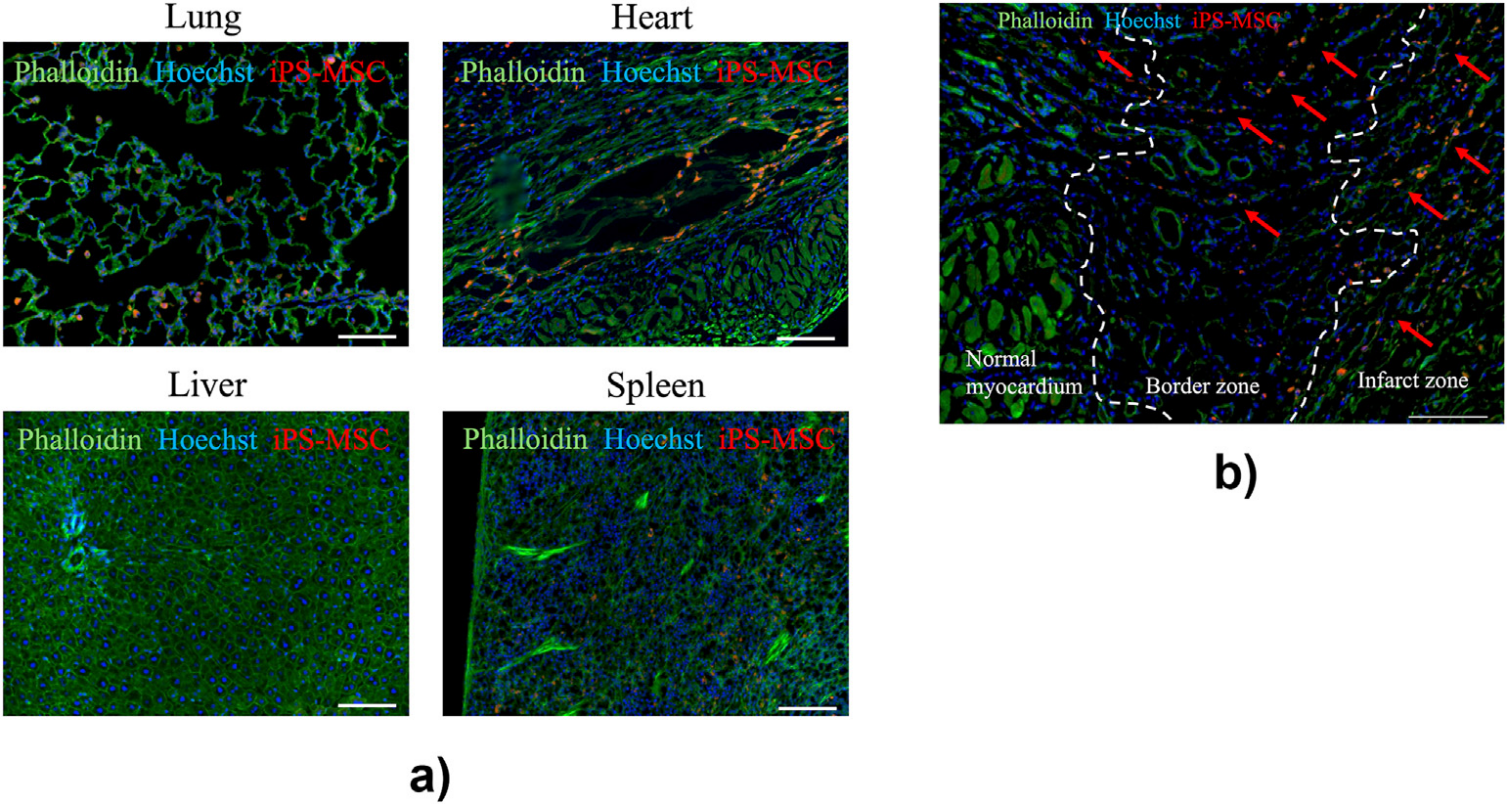
**Distribution and localization of iPS-MSCs in organs after systemic administration.** (a) Representative images of fluorescence staining with CellTracker probes in the lung, heart, liver, and spleen. Scale bar, 100 μm. (b) Representative images of fluorescence staining with a focus on the border zone of the myocardium. Red arrows indicate CellTracker-positive iPS-MSCs. The picture showed iPS-MSCs accumulation in the vasculature of the border zone and migration from the normal myocardium to the infarct zone. Scale bar, 100 μm.

The distribution of the administered iPS-MSCs was evaluated using cell-tracker fluorescent dyes during the acute phase. Immunofluorescence staining of rat organ tissues revealed iPS-MSC accumulation, mainly in the lung vasculature, 24 h after systemic cell administration (Fig. 4a). Some cells accumulated in the spleen and a few cells were observed in the liver. In the heart, iPS-MSCs accumulate around the infarct border zone. A detailed picture of the heart demonstrated that iPS-MSCs migrated from the normal myocardium to the infarct zone and were mostly distributed in the infarct border zone vasculature (Fig. 4b).

### 3.6 iPS-MSCs induce anti-inflammatory response and immunomodulation effect in rat heart tissue

**Fig. 5:**
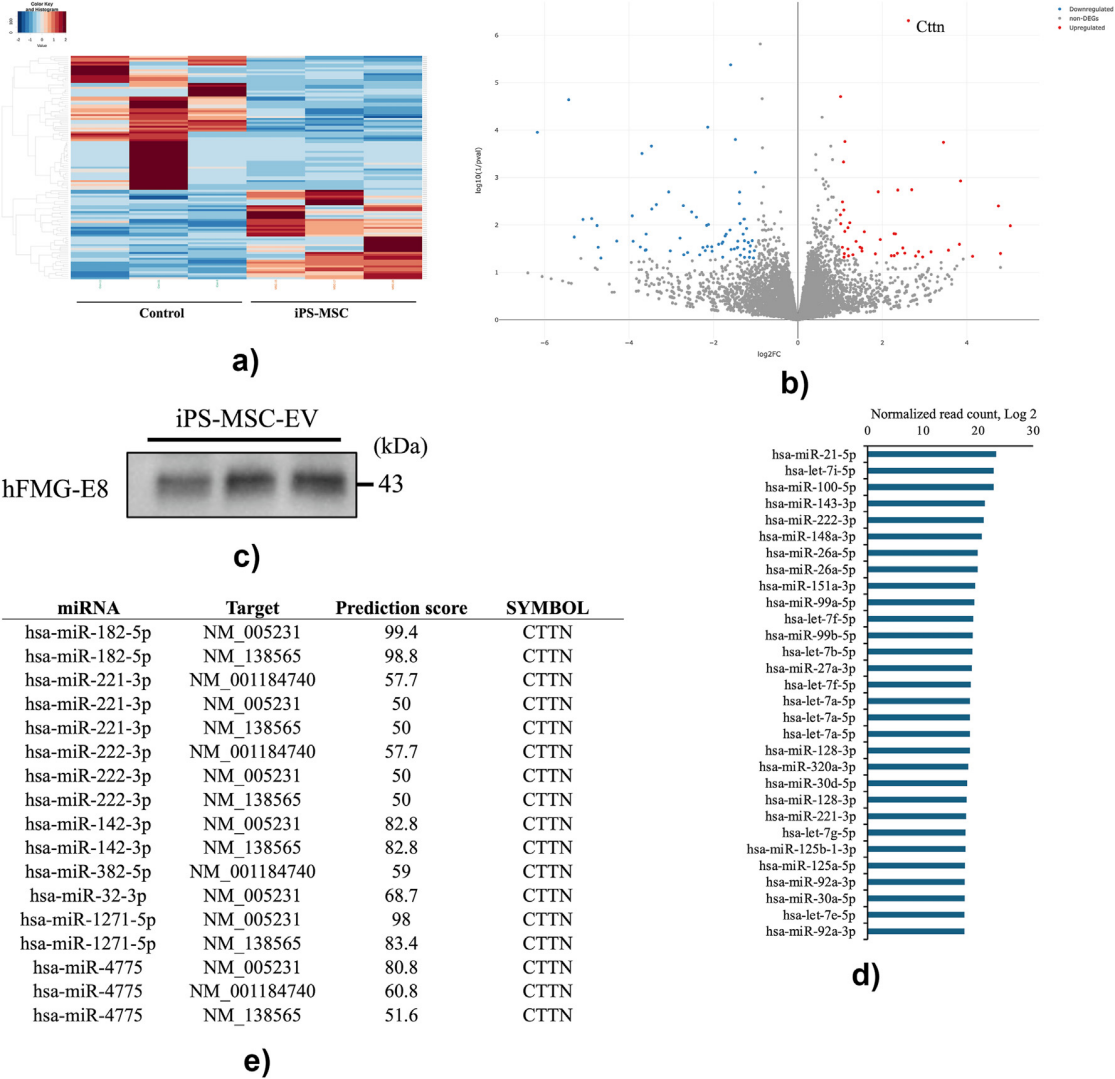
**Comprehensive RNA-sequencing analysis in the border zone of rat myocardium and microRNA sequencing analysis of iPS-MSC extracellular vesicles.** (a) Heatmap showing the expression values of differentially expressed genes (DEGs) in the iPS-MSC group compared with the control group. (b) Volcano plot showing an overview of the DEGs. The log2 fold change was plotted on the x-axis and the negative log10 (1/pval) was plotted on the y-axis. The red dots show single genes with significant increases and the blue dots show single genes with significant decreases. (c) EVs from the medium were subjected to western blotting using antibodies against EV marker proteins. (d) The top 30 most abundant miRNAs from the comprehensive miRNA sequencing data are listed in the table. (e) miRNA target prediction based on matched data from RNA sequencing analysis of the rat myocardium. Eight miRNAs and their target mRNAs are shown in the table. miRNA, microRNA; EV, extracellular vesicles.

Total RNA was extracted from the infarct border zone of the rat myocardial tissue and analyzed through comprehensive RNA sequencing. The heat map shows 46 upregulated and 69 downregulated differentially expressed genes (DEGs) in the iPS-MSC group compared with the control group (Fig. 5a). The volcano plot shows an overview of the differentially expressed genes by comparing the iPS-MSCs and control groups (Fig. 5b). *CTTN*, the gene labeled in the figure, was significantly upregulated in the iPS-MSC group. The MA plot shows the relationship between the average concentration (log (baseMean)) and the fold change (log2 FC) across single genes (Fig. S1). The Gene Ontology (GO) enrichment analysis was performed and visualized using a graphical structure (Fig. S2). Fig. S2 lists the enriched GO terms in biological processes, which were separated into upregulated and downregulated genes. Analysis of upregulated genes revealed enrichment in GO terms associated with cell migration during sprouting angiogenesis (GO: 0090050), negative regulation of apoptosis (GO: 1902257), and positive regulation of vascular wound healing (GO: 0035470). Among the GO terms enriched for the downregulated genes, inflammatory response (GO: 0006954), CD8-positive T cell differentiation (GO: 0002305 and GO: 0002300), and cytotoxic T cell differentiation (GO: 0045065) were identified.

### 3.7 iPS-MSC extracellular vesicle isolation and microRNA analysis

EVs were isolated from a serum-free culture medium of iPS-MSCs using modified polyethylene glycol and ultracentrifugation [17]. The Western blot analysis of iPS-MSC EVs confirmed the highly expressed exosome marker protein hMFG-E8 (Fig. 5c). MicroRNAs were extracted, and a comprehensive miRNA sequencing analysis was performed. The top 30 miRNAs are shown in Fig. 5d. The list includes miRNAs involved in angiogenesis (has-miR-21–5p, has-miR-122–5p, and has-miR-423–5p) [[22], [23], [24]], anti-inflammation (has-miR-222–3p) [25], and cardiac development and myogenesis (has-miR-143–3p and has-miR-27a-3p) [26,27]. MicroRNA target prediction based on matched data from comprehensive RNA sequencing of heat tissues was performed. Fig. 5e shows the eight miRNAs and their target mRNAs, suggesting that these miRNAs regulate CTTN as a target gene that encodes cortactin, a protein involved in the actin cytoskeleton, cell migration, and intracellular transport [28].

## 4 Discussion

This study demonstrated that systemic sequential iPS-MSC administration improved cardiac function in an ICM rat model, attenuated cardiac fibrosis, and enhanced angiogenesis in the border zone of the myocardium. Thus, EVs and their miRNAs may play important roles in the cardioprotective and immunomodulatory functions of iPS-MSCs. Three pieces of evidence from our experiments supported this finding. First, although iPS-MSC administration provided cardioprotective and anti-fibrotic effects with ICM, these therapeutic effects were significantly mitigated by the knockdown of Alix, which is a key protein in exosome biogenesis. Second, the administered cells accumulated in the border zone of the ischemic myocardium according to cell-tracking analysis, suggesting that the homing ability to the damaged tissue site plays a crucial role in the biomechanism of MSC. Third, in the miRNA sequencing analysis, EVs released from iPS-MSCs were rich in crucial miRNAs involved in angiogenesis, anti-fibrosis, and anti-apoptosis.

Administered MSCs can be recruited into the bloodstream and migrate to the injury site by the guidance of various chemotactic signals [[29], [30], [31]]. Preclinical and clinical studies showed EVs administered in systemic circulation provide disease-modifying cardioprotective effects [32,33]. Those therapeutic effects are presumably caused by the interaction with the endogenous immune system, mainly by the actions of monocytes and macrophages [34]. Moreover, EVs released from MSCs have a paracrine effect on surrounding tissues; therefore, locally accumulated MSCs would release EVs and carry angiogenetic and anti-fibrotic effects to adjacent myocardium and other cells [35]. EVs released from MSC can transmit their information to target cells through encapsulated contents, such as miRNA and protein, resulting in modification of target cell activity and function [36].

EVs from iPS-MSCs have similar biological characteristics and therapeutic effects as iPS-MSCs alone. A locally hypoxic environment would also accelerate EV release from cells and modify the characteristics of their miRNA cargo [37]. miRNAs in EVs from MSCs play a key role in regulating inflammation by reducing pro-inflammatory cytokines and transforming macrophages from the M1 to M2 anti-inflammatory phenotype [[38], [39], [40]]. EVs released from the cell can affect various pathways involved in angiogenesis, antifibrosis, and immunomodulation through the miRNA-regulatory network. miR-21–5p and miR-222–3p play an important role in anti-inflammation through the degradation of the target gene *CCR7* [41].

EVs from MSCs also accelerate angiogenesis through the upregulation of IGF-1α and pAkt [42]. Moreover, GO term enrichment analysis of the differentially expressed genes of the rat heart tissue in the iPS-MSC group revealed upregulation of specific pathways regarding positive cell migration regulation, platelet-derived growth factor (PDGF) production, and vascular wound healing, as well as negative apoptotic process regulation. PDGF is one of the key growth factors involved in MSC migration [10]. These upregulated pathways suggest that administered MSCs interact with the heart tissue, enable cell migration to the border zone of the myocardium, and promote tissue repair [31]. The downregulated pathways regarding positive regulation of T cell differentiation and activation suggest the immunomodulatory effects of the iPS-MSCs, which enable cell engraftment into the heart tissue. This finding indicates that MSC derived from human iPSC can engraft the rat ICM model without using immunosuppressants through their immunomodulatory effects. Furthermore, the target prediction of upregulated miRNAs suggests that the CCTN gene is a target gene. CCTN encodes cortactin, an actin-binding protein that contributes to the organization of the cytoskeleton and cell morphology [28]. Cortactin also plays a major role in cell migration, intracellular transport, and signaling [43]. The results of the miRNA target analysis suggest that cellular communication through the miRNA network influences the migration and homing of MSCs to the injury site.

This study has some limitations. Although we focused on miRNAs as key factors of EV components involved in the therapeutic effects of iPS-MSCs, the functional mechanisms of other EV elements, including proteins and lipids, are unknown. We also performed target prediction based on the miRNA analysis. However, its detailed mechanism of action in cardiac tissue remains unclear. Furthermore, we were unable to determine the optimal iPS-MSC dosage. Further research is required to address these issues.

## 5 Conclusions

The systemic iPS-MSC administration improved cardiac function through local accumulation at the injury site, angiogenesis, and antifibrotic effects in a rat ICM model, suggesting the clinical possibility of treating chronic heart failure. EVs and their miRNA cargoes play key roles in the therapeutic effects of iPS-MSCs through local paracrine action.

## Data availability statement

All data generated or analyzed in this study are available in the article and supplemental materials.

## Funding

This study was supported by a Japan Society for the Promotion of Science KAKENHI Grant (T23K082530).

## Declaration of competing interest

The authors declare that they have no known competing financial interests or personal relationships that could have appeared to influence the work reported in this paper.
