# Supplementary material for: Systemic administration of induced pluripotent stem cell-derived mesenchymal stem cells improves cardiac function through extracellular vesicle-mediated tissue repair in a rat model of ischemic cardiomyopathy
Source: Regen Ther. 2024 Dec 31;28:253–61. doi: 10.1016/j.reth.2024.12.008 (PMC11745812; doi:10.1016/j.reth.2024.12.008)

**Supplementary Material**

**Legend for Supplementary Figures**

**Figure S1. MA plot of the differentially expressed genes.**

The MA plot shows the relationship between the average concentration (log (baseMean)) and fold change (log2 FC) across the single genes in the iPS-MSC group compared with the control group. The red dots represent significantly upregulated differentially expressed genes, and the blue dots represent significantly downregulated differentially expressed genes.

**Supplementary Figure S2. Gene ontology enrichment analysis in biological process**

(a) Genes upregulated in gene ontology enrichment analysis of biological processes. (b) Downregulated genes in gene ontology enrichment analysis of biological processes.

**Figure S1**


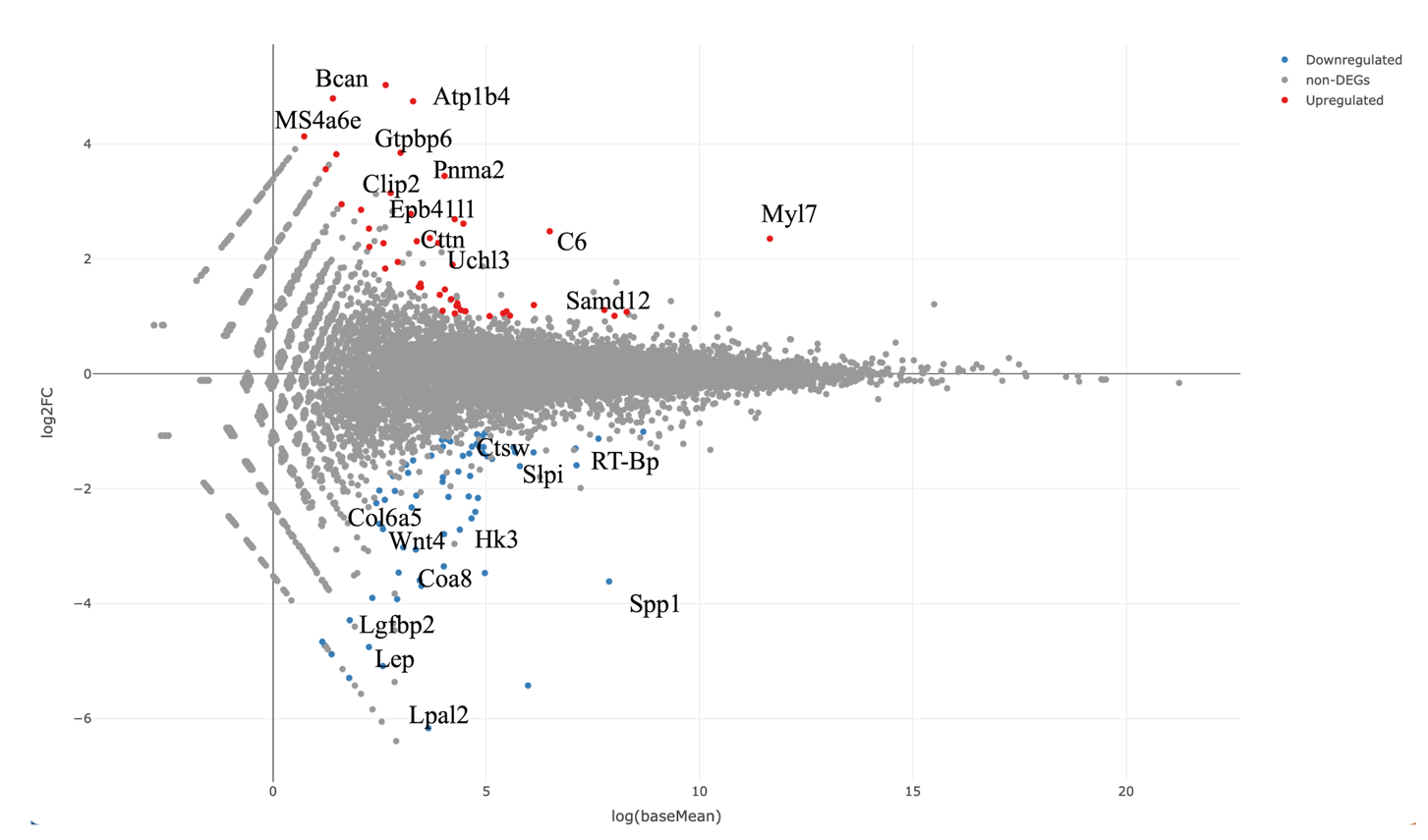


**Figure S2 (a)**


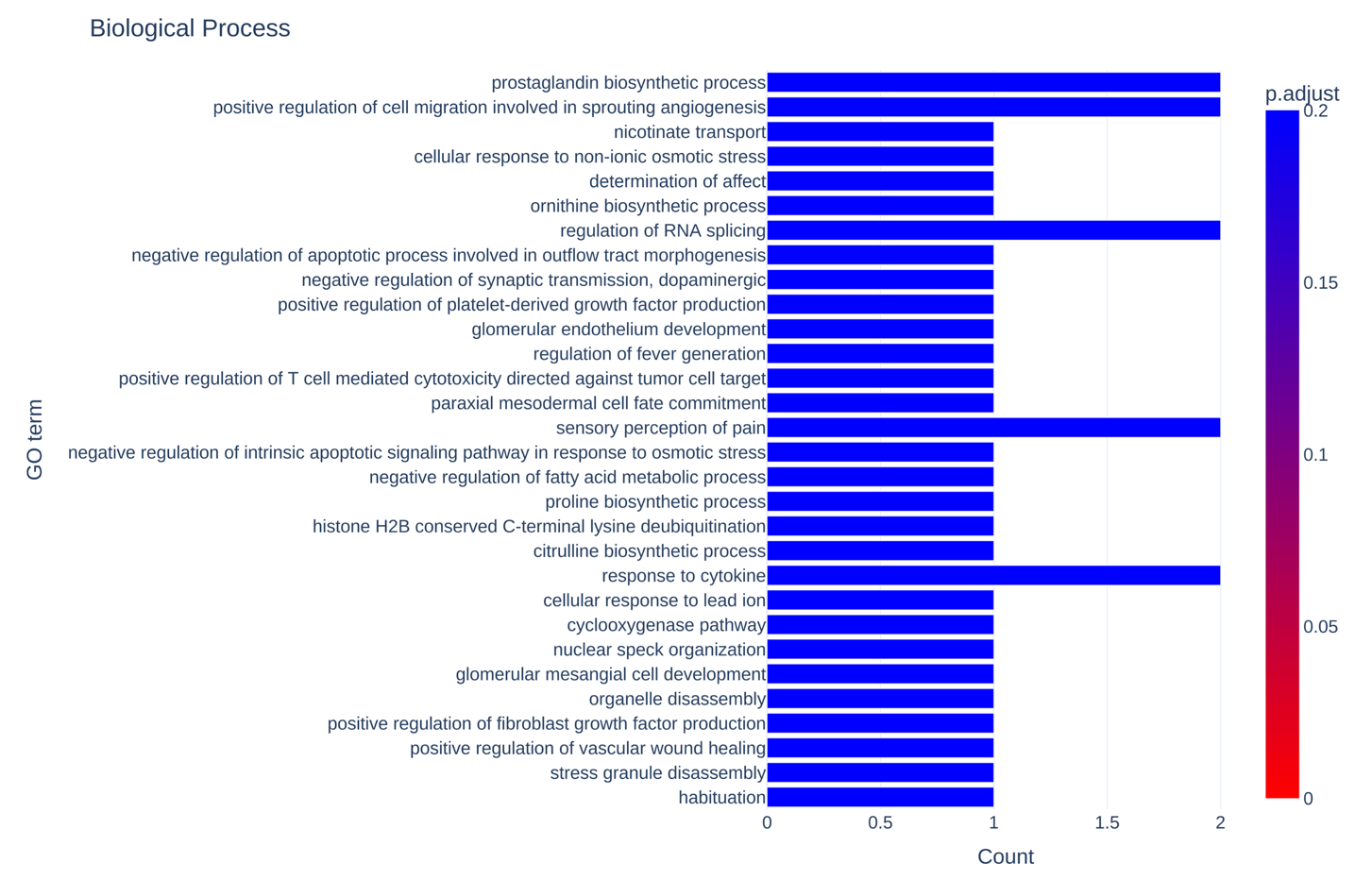


**Figure S2 (b)**


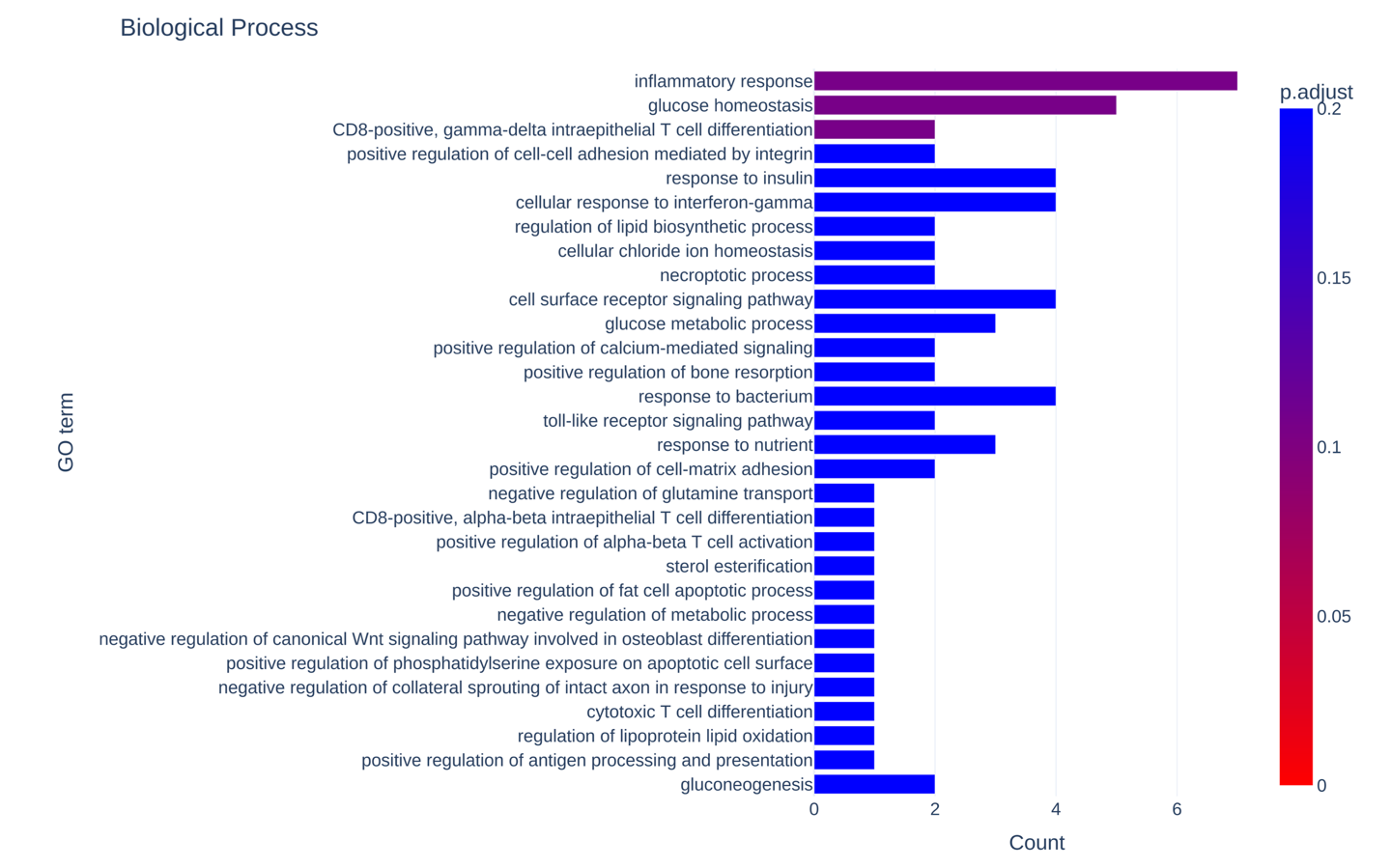

Supplement: Multimedia component 1 [file mmc1.docx]
